# Novel minimally invasive tape suture osteosynthesis for instabilities of the pubic symphysis: a biomechanical study

**DOI:** 10.1007/s00402-021-03968-z

**Published:** 2021-05-29

**Authors:** Adrian Cavalcanti Kußmaul, Fanny Schwaabe, Manuel Kistler, Clara Gennen, Sebastian Andreß, Christopher A. Becker, Wolfgang Böcker, Axel Greiner

**Affiliations:** 1 Department of General Trauma and Reconstructive Surgery, University Hospital LMU Munich, Munich, Germany; 2 Department of Orthopedics, Physical Medicine and Rehabilitation, University Hospital LMU Munich, Munich, Germany; 3 Department of Obstetrics and Gynecology, University Hospital LMU Munich, Munich, Germany

**Keywords:** Pelvic instability, Biomechanics, Minimally invasive, Flexible osteosynthesis, Pubic symphysis, SpeedBridge^™^

## Abstract

**Introduction:**

Open book fractures are challenging injuries oftentimes requiring surgical treatment. The current treatment of choice is symphyseal plating, which requires extensive surgery and entirely limits physiological movement of the symphyseal joint, frequently resulting in implant failure. Therefore, we investigated the biomechanical properties of a semi-rigid implant (modified SpeedBridge^™^) as a minimally invasive tape suture construct for the treatment of open book fractures and evaluated the superiority of two techniques of implementation: criss-cross vs. triangle technique.

**Materials and methods:**

Nine synthetic symphyseal joints were dissected creating an open book fracture. The different osteosynthesis methods (plating, modified SpeedBridge^™^ in criss-cross/triangle technique) were then applied. All constructs underwent horizontal and vertical loading, simulating biomechanical forces while sitting, standing and walking. For statistical analysis, dislocation (mm) and stiffness (N/mm) were calculated.

**Results:**

Symphyseal plating for the treatment of open book fractures proved to be a rigid osteosynthesis significantly limiting the physiological mobility of the symphyseal joint (dislocation: 0.08 ± 0.01 mm) compared to the tape sutures (dislocation: triangle technique 0.27 ± 0.07 mm, criss-cross technique 0.23 ± 0.05 mm) regarding horizontal tension (*p* < 0.01). Both modified SpeedBridge^™^ techniques showed sufficient biomechanical stability without one being superior to the other (*p* > 0.05 in all directions).

Considering vertical loading, no statistical difference was found between all osteosynthesis methods (caudal: *p* = 0.41; cranial: *p* = 0.61).

**Conclusions:**

Symphyseal plating proved to be the osteosynthesis method with the highest rigidity. The modified SpeedBridge^™^ as a semi-rigid suture construct provided statistically sufficient biomechanical stability while maintaining a minimum of symphyseal movement, consequently allowing ligamental healing of the injured joint without iatrogenic arthrodesis. Furthermore, both the criss-cross and the triangle technique displayed significant biomechanical stability without one method being superior.

## Introduction

Open book fractures of the pelvic ring are challenging injuries oftentimes caused by high impact trauma [1]. They are usually based on an anteroposterior pelvic compression resulting in the tearing of the symphysis [2].

According to the AO comprehensive classification, an open book fracture is classified as a type 61-B2 or type 61-B3 fracture, depending on the location and the severity of the involvement of the posterior pelvic ring [3].

Surgical treatment, including anatomical reduction and adequate fixation, of the anterior pelvic ring contributing approximately 30% to the stability of the entire pelvic ring [4] is necessary to restore biomechanical stability and to ensure an adequate healing of the symphysis and an early mobilisation of the patient [1, 5].

Currently, open reduction and internal fixation with a multi-hole plate has largely been accepted to be the gold standard [6], inhibiting mobility of the symphysis by consequently maintaining diastatic reduction [7].

However, critics emphasise that the symphysis as a cartilaginous joint displays a physiological movement up to 2 mm [8]. This movement is consequently compromised by rigid fixation systems potentially resulting in implant failure and the need for operative revision [1, 8–10]. Also, this technique requires extensive surgery with numerous potential complications, such as high blood loss, lesion of surrounding neurovascular structures and internal organs and oftentimes hardware removal after 6 months [1, 11, 12].

To address these issues, several approaches have been investigated using numerous internal fixation methods such as cerclages and self-degrading Polydioxanon (PDS)-bandings [13], internal fixators [14] and two-hole plates [11, 15]. Yet, these methods also display a high rate of implant failure based on an insufficient fixation of the symphyseal joint prior to a ligament healing [13, 16].

Recently, tape sutures such as the SpeedBridge^™^ (Arthrex, Naples, Florida, USA) have displayed promising results in the surgical treatment of weight-bearing joints like the syndesmosis of the ankle joint [17] and ligamental injuries of the rotator cuff or the knee [18, 19]. In a recent study, the FiberTape^®^ (Arthrex, Naples, Florida, USA) has also been successfully used for the fixation of incomplete posterior fractures of the pelvic ring [20].

The biomechanical properties of these semi-rigids implants are based on the reinforcement of ligamental structures and the associated splinting of the injury, avoiding an unphysiological arthrodesis of the symphysis without affecting ligamental healing [20]. A further advantage is their minimally invasive insertion with concomitant tissue protection [20].

Therefore, the aim of this biomechanical study was to examine the feasibility and biomechanical properties of semi-rigid implants (modified SpeedBridge^™^) for the treatment of open-book fractures based on the following two questions:

- Does the investigated tape suture construct (modified SpeedBridge^™^) provide sufficient biomechanical stability of the symphysis while simultaneously avoiding an unphysiological arthrodesis of the symphyseal joint when using a multi-hole plate?
- Is a criss-cross technique using four anchors biomechanically superior to a triangle technique using three anchors?

## Materials and methods

**Fig. 1:**
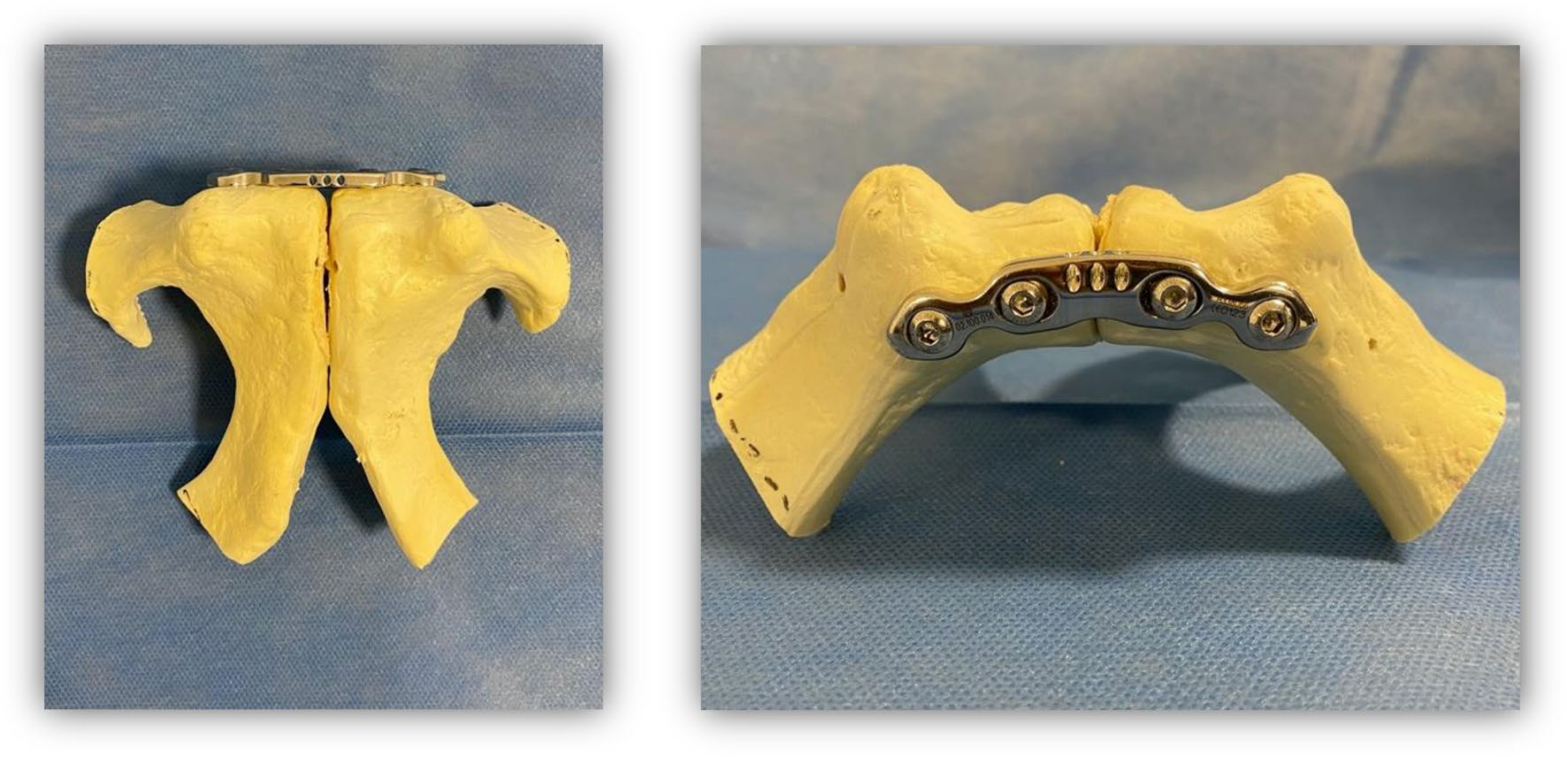
Isolated pubic symphysis with plate fixation

**Fig. 2:**
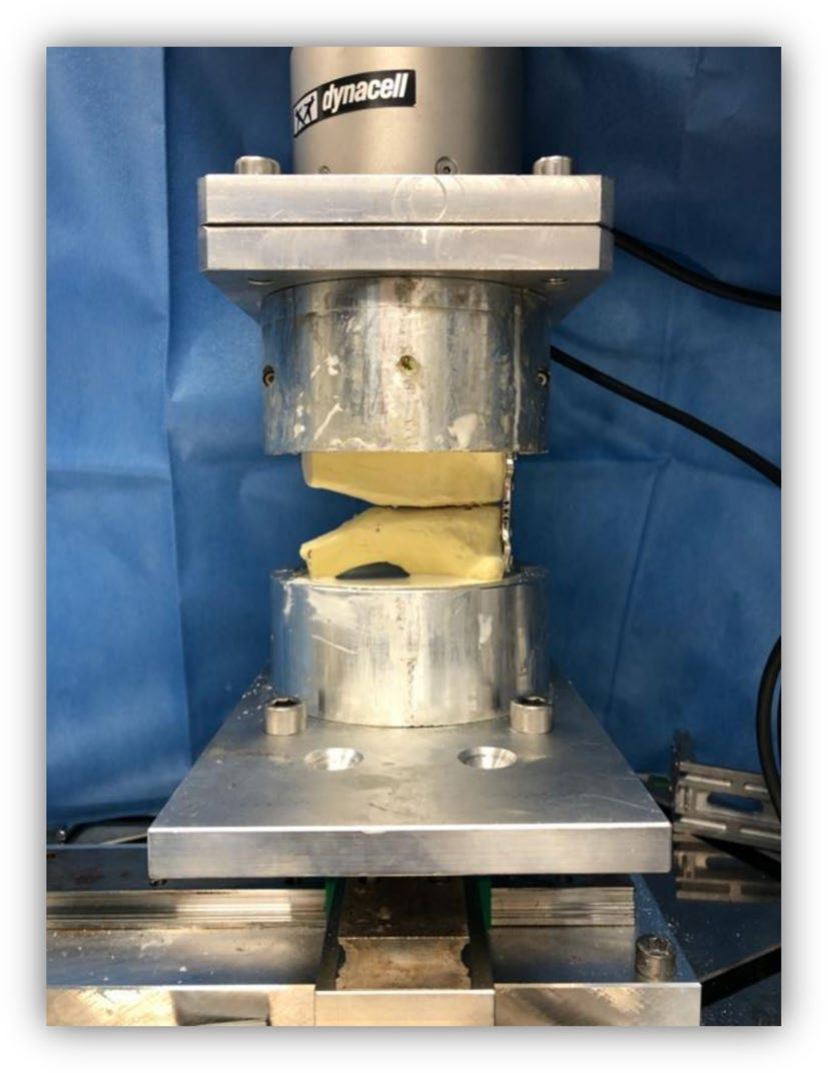
Isolated symphysis with plate fixation embedded and mounted on biomechanical testing machine (Instron ElectroPuls^™^ E10000 Linear-Torsion)

A total of nine composite synthetic full pelvises (Model: Full Pelvis 1301, Sawbones^®^; Pacific Research Laboratories, Vashon, WA, USA) were used in this study. To isolate the symphysis, the rami superiors and inferiors of the pubic bone were cut out on the lateral side of the obturator ring using a hacksaw. For the creation of an open-book fracture, the interpubic disc was dissected, resulting in the complete separation of both sides. For the first trial, all specimens were fixated with a symphyseal plate (DePuySynthes 3.5; four holes, dynamic compression plate) and four identical cortical screws (DePuySynthes Cortex Screw 3.5 mm, 50 mm) (see Fig. 1). The pelvises were then symmetrically embedded into metal cylinders for experimental testing (see Fig. 2).

**Fig. 3:**
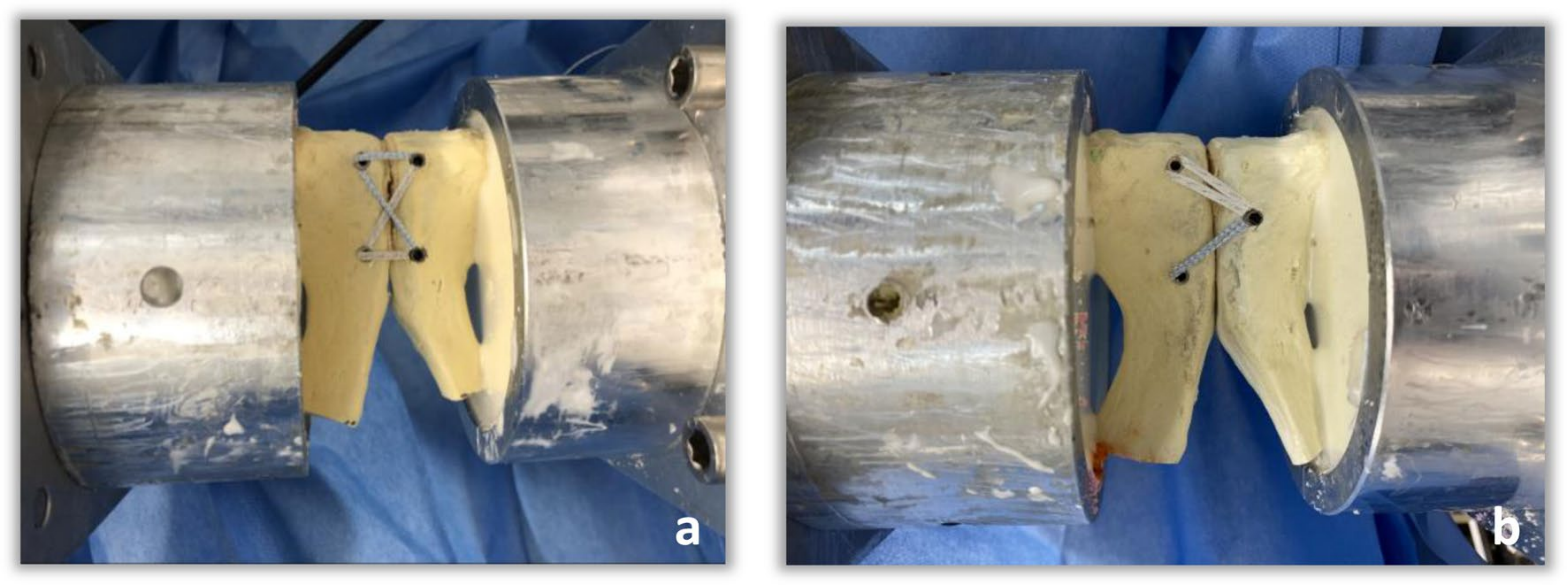
Modified SpeedBridge™ in criss-cross **a** and triangle **b** technique

Next, the plate osteosynthesis was removed and the modified SpeedBridge^™^ (Arthrex, Naples, FL, USA) was applied to the pelvises (see Fig. 3). Two different tape suture configurations were used: a criss-cross technique and a triangle technique. For the criss-cross technique, a total of four holes were drilled into the pubic bone laterally to the symphysis as shown in Fig. 3a.

The right holes were drilled with a 3.5 mm drill and an Arthrex (Naples, FL, USA) PEEK SwiveLock^®^ 4.5 mm anchor armed with a FiberTape^®^ was inserted in each hole. The tapes were then tightened in a criss-cross technique and were each fixated on the left side with a 6.5 mm titan Corkscrew^®^. The left hand-sided holes were pre-drilled with a 4.5 mm drill.

For the triangle technique, two holes were drilled on the right side of the symphysis with a 3.5 mm drill identically to the criss-cross technique, here, however, with only one hole being drilled on the left side with a 4.5 mm drill (see Fig. 3b). The Tapes coming from the right side were then spanned to the left and fixed with a 6.5 mm titan Corkscrew^®^.

To reduce bias within the study, the sequence of the suture technique (criss-cross technique or triangle technique) applied first was randomly allocated for each specimen.

After each tape suture fixation, the pelvises underwent biomechanical testing according to a standardised protocol (see Table 1).

**Table 1:** 8-step protocol for biomechanical testing

Step 1	Compression force loading up to −50 N
Step 2	Holding at −50 N for 30 s
Step 3	Periodic loading: 30 cycles with a frequency of 1 Hz between −75 N and −25 N
Step 4	Decrease loading to 0 N
Step 5	Tractive Force loading up to 50 N
Step 6	Holding at 50 N for 30 s
Step 7	Periodic loading: 30 cycles with a frequency of 1 Hz between + 75 N and + 25 N
Step 8	Decrease loading to 0 N

The pelvises were mounted into an all-electric industrial loading machine (Instron ElectroPuls^™^ E10000 Linear-Torsion, Norwood, MA, USA) (see Fig. 2) and an 8-step testing protocol based on Meissner et al [13] (see Table 1) was applied. In this study, Meissner et al initially tried to simulate full body weight on an isolated symphysis which, however, led to the failure of all implants [13]. Ultimately, the original forces were then cut in half, leading to realistic biomechanical results [13] and finally preparing the ground for the forces used in this experimental study.

**Fig. 4:**
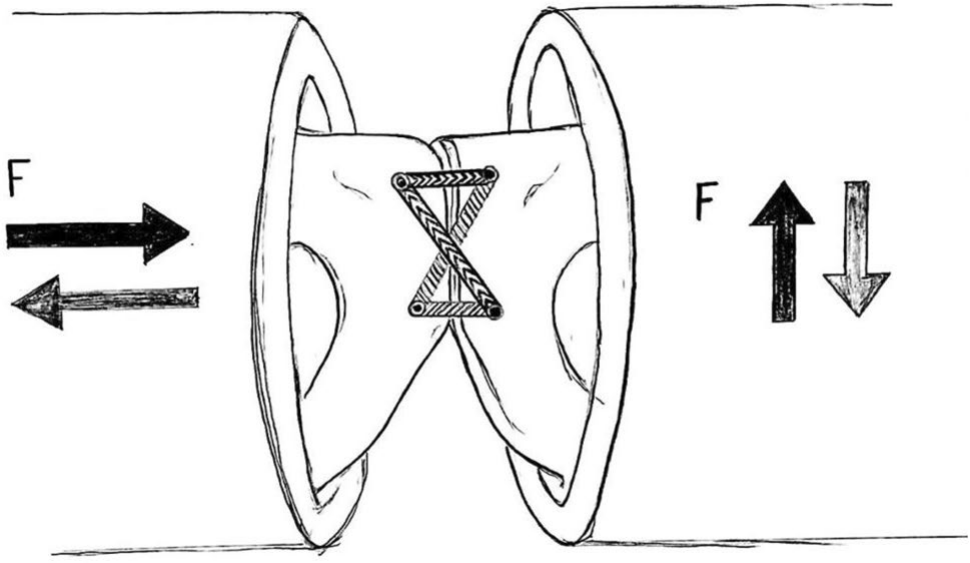
Direction of horizontal and vertical loading forces (F)

The specimens were loaded both horizontally and vertically, simulating the forces effecting the symphyseal joint while sitting, standing and walking (see Fig. 4).

Based on previous research [20, 21], the parameters displacement (mm) and stiffness (N/mm) were used to compare the biomechanical properties of the different fixation techniques.

The displacement was defined as the average maximum displacement (mm) between the fracture fragments during cyclic loading, whereas the stiffness (N/mm) was defined as the maximal force during the cyclic movement (50 N) divided by the maximum displacement (mm). Both parameters were analysed separately for all directions (vertical and horizontal, compression and tension).

During the application of these forces, the displacement (mm) of the symphysis was continuously measured and the stiffness (N/mm) consecutively calculated.

During the transition of the pelvises from horizontal to vertical, the metal cylinders were fixed by a rod preventing any force affecting the symphyseal joint or the fixation method which could possibly interfere with the experiment.

The statistical analysis was performed with IBM SPSS Statistics (Windows, version 26.0, IMB Corp., Armonk, NY, USA). Based on data presented by Meissner et al [13], we estimated that nine specimens would yield 94% power at the 5% significance level.

Normal distribution of variables was determined by performing the Kolmogorov–Smirnov and Shapiro–Wilk tests. In case of divergent results, the data were analysed both as normally distributed and as non-normally distributed and compared afterwards.

Quantitative variables were compared by either a two-tailed *T* test for normally distributed or a Mann–Whitney *U* test for not normally distributed variables. Analysis of variance (ANOVA) or a Kruskal–Wallis test was used when comparing more than two groups.

## Results

**Fig. 5:**
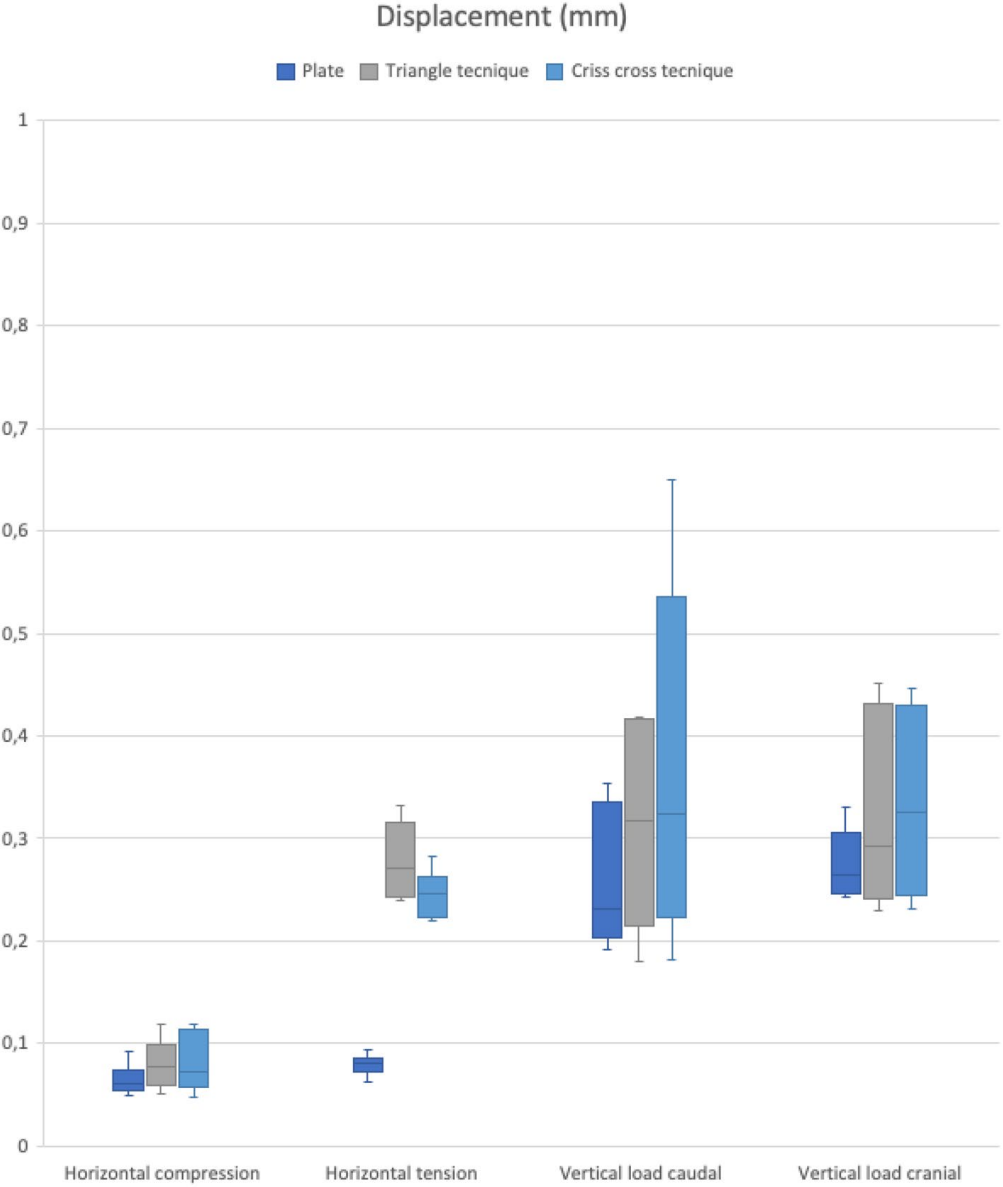
Triangle technique

**Fig. 6:**
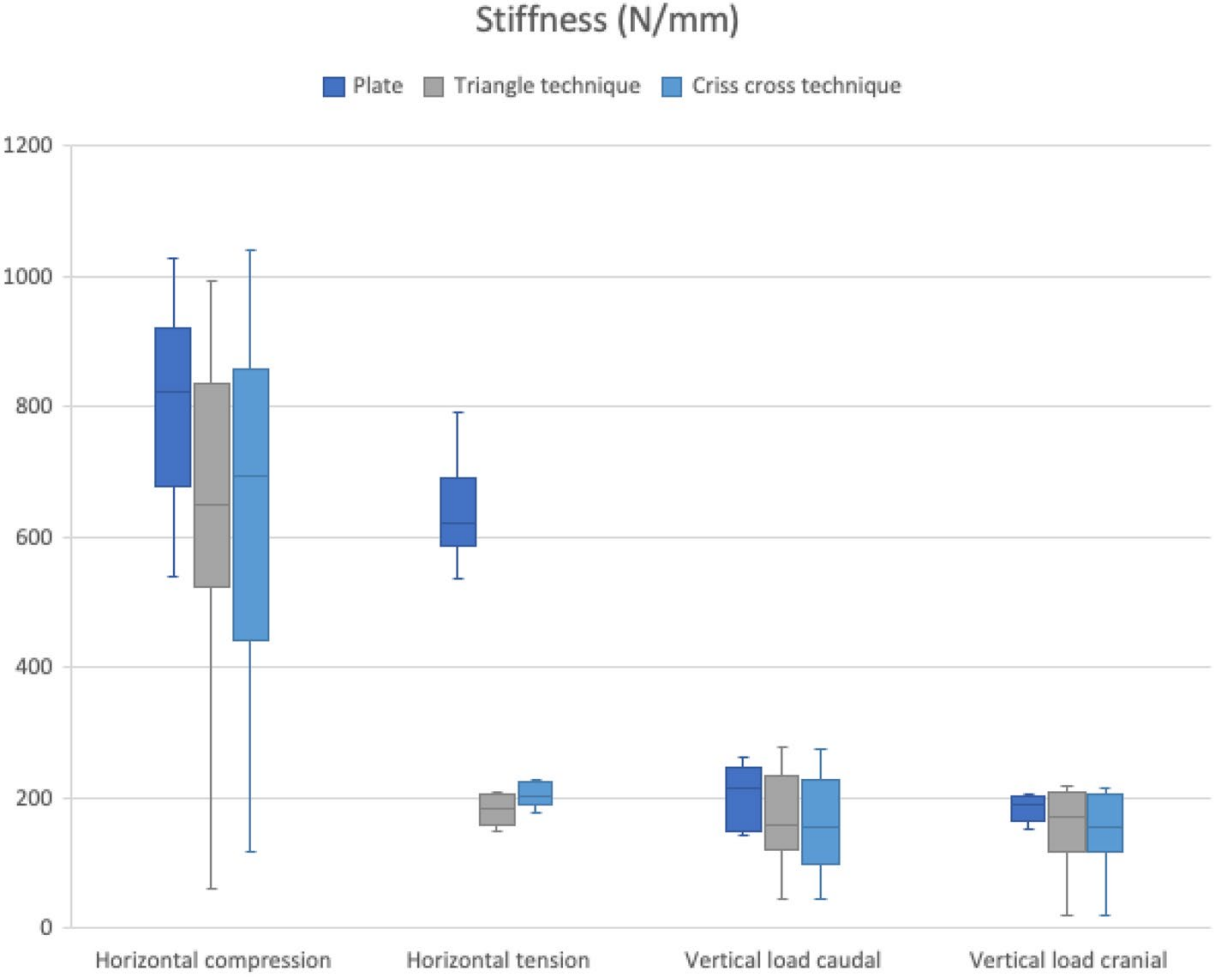
Criss cross technique

Comparing the mean displacement and the mean stiffness as well as the median between the different groups, plate fixation demonstrated the highest stiffness and the least displacement between all techniques in all directions (see Tables 2, 3 and Figs. 5, 6). The highest average dislocation was found for the triangle technique in the horizontal direction (0.27 ± 0.07 mm) and for the criss-cross technique in the vertical direction (0.57 ± 0.76 mm) (see Tables 2, 3 and Figs. 5, 6).

**Table 2:** Mean displacement (mm)

	Horizontal loading		Vertical loading	
	Compression	Tension	Caudal	Cranial
Plate group	0.06 (± 0.01)	0.08 (± 0.01)	0.26 (± 0.06)	0.27 (± 0.03)
Triangle group	0.16 (± 0.25)	0.27 (± 0.07)	0.39 (± 0.30)	0.56 (± 0.76)
Criss-cross group	0.11 (± 0.12)	0.23 (± 0.05)	0.42 (± 0.29)	0.57 (± 0.76)
Difference between the groups	*p* = 0.24	*p* < 0.01	*p* = 0.41	*p* = 0.61

**Table 3:** Mean stiffness (N/mm)

	Horizontal loading		Vertical loading	
	Compression	Tension	Caudal	Cranial
Plate group	806.75 (± 153.92)	641.21 (± 78.45)	204.07 (± 46.87)	184.24 (± 20.29)
Triangle group	637.27 (± 270.34)	216.20 (± 119.38)	171.94 (± 74.70)	154.41 (± 63.93)
Criss-cross group	657.76 (± 280.72)	227.73 (± 78.08)	158.70 (± 76.72)	149.78 (± 62.55)
Difference between the groups	*p* = 0.29	*p* = 0.10	*p* = 0.36	*p* = 0.34

When comparing the mean displacement of plate osteosynthesis (0.08 ± 0.01 mm) to each tape suture technique (triangle technique: 0.27 ± 0.07 mm, criss-cross technique: 0.23 ± 0.05 mm) during horizontal tension, a significant difference was found (*p* < 0.01), while there was no significant difference between the triangle and the criss-cross techniques (*p* = 0.30). No significant difference between fixation techniques was detected either during horizontal (lateral) compression (*p* = 0.24) or vertical loading (cranial: *p* = 0.61, caudal: *p* = 0.41), (see Table 2 and Fig. 5).

A direct comparison of the mean stiffness under tension in the horizontal direction indicated a significant difference both between the plate and the triangle techniques (*p* < 0.01) and between the plate and the criss-cross techniques (*p* < 0.01). A direct comparison of the tape techniques was found to be non-significant (*p* > 0.05 in all directions).

## Discussion

Open book fractures of the pelvic ring are severe injuries that normally require surgical intervention to restore pelvic stability and to consequently maintain the patient’s mobility [1, 22, 23].

Currently, the widely preferred treatment method is open reduction and internal fixation with a multi-hole angle-stable plate osteosynthesis [22, 24]. However, there are some major limitations this rigid osteosynthesis technique demonstrates. First, it compromises the physiological mobility of the symphyseal joint, frequently resulting in implant failure in up to 43% of cases, loss of reduction in up to 24% and the need for operative revision in up to 9% [11, 15, 16, 22, 24–26]. Secondly, the extent of the procedure’s invasiveness is associated with numerous surgical complications, such as increased blood loss and the endangerment of the surrounding structures [1, 11]. Third, many patients later require or wish a removal of the inserted plate, oftentimes due to comfort, psychological and obstetric reasons or complete recovery with the idea of preventing possible plate-associated complications [24].

In our biomechanical study, we could confirm that symphyseal plating as a rigid osteosynthesis with regard to a physiological mobility of the symphyseal joint of up to 2 mm [8] almost entirely compromised the mobility of the symphyseal joint (see Table 2 and Fig. 5). These findings correspond with the current literature identifying symphyseal plating as an osteosynthesis method with sufficient biomechanical stability based on the fixation of the symphyseal joint [27, 28].

With regard to the first question of this study, we found that the modified SpeedBridge^™^ provides sufficient biomechanical stability for the treatment of open book fractures of the pelvis without a single failure in all nine pelvic models.

Considering the standard deviation of the dislocation of the tape suture constructs, the maximum dislocation was found for the criss-cross technique during vertical cranial loading with 0.57 ± 0.76 mm. Even this dislocation with a maximum of 1.33 mm approaches but still does not exceed the physiological movement of the symphysis of up to 2 mm [8].

Also, under horizontal tension, we found a significant difference between the plate osteosynthesis and the tape suture techniques (*p* < 0.01). With a mean dislocation of 0.27 ± 0.07 mm (triangle technique) and 0.23 ± 0.05 mm (criss-cross technique), both tape sutures proved to provide sufficient biomechanical stability allowing some yet not exceeding the physiological mobility of the symphyseal joint, while the plate osteosynthesis with a dislocation of 0.08 ± 0.01 mm almost completely prohibits any symphyseal movement.

Consequently, we could demonstrate that the tape suture constructs provide sufficient biomechanical stability for ligamental healing while maintaining a minimum of symphyseal mobility preventing an iatrogenic arthrodesis of the symphyseal joint.

Considering horizontal compression, we did not find a significant difference between all osteosynthesis methods (*p* = 0.24), most likely due to the self-limitation of the symphyseal bony structure (see Fig. 1).

Also non-significant, but still biomechanically relevant, were the findings of the dislocation of the osteosynthesis methods during vertical loading (cranial: *p* = 0.61, caudal: *p* = 0.41). When applying vertical load to the plate osteosynthesis, the plate allows three times more micromovements (cranial: 0.27 ± 0.03 mm, caudal: 0.26 ± 0.06 mm) than under horizontal forces (< 0.1 mm), in our opinion possibly favouring implant failure. In contrast, even though the flexible implants allowed more movement (see Table 2), they did neither exceed the assumed physiological symphyseal mobility [8] nor loosen in any of the tested pelvises, supporting our hypothesis of sufficient biomechanical stability without compromising the symphyseal joint.

Taking the stiffness into account and based on the mathematical definition, the findings of the stiffness corresponded to the results of the dislocation (see Tables 2, 3 and Figs. 5, 6).

It is also worth mentioning that the plate displayed a significantly greater stiffness than both the triangle and the criss-cross technique (both *p* < 0.01), again underlying the unphysiological compromising of the symphyseal joint by the plate osteosynthesis and emphasizing the biomechanical stability of the tape suture constructs.

Supporting our evidence, Kiskaddon et al likewise reported a sufficient biomechanical stability of a nitinol wire construct (TightRope^®^, Arthrex, Naples, FL) when compared to a symphyseal plate [29]. However, these authors used an endobutton technique for the fixation of the TightRopes^®^ on the posterior symphyseal surface [29]. This technique requires the surgical access to the posterior side of the symphyseal joint, potentially irritating or even endangering the urinary bladder and the surrounding tissue when implanting the endobuttons [29]. In contrast, the technique used in this study uses bone anchors attached to the anterior side of the symphysis only, consequently without the necessity of posterior preparation. In our opinion, this allows the clinical implementation of the tape construct as a minimal invasive procedure preserving soft tissue without constraints of the biomechanical properties.

Arner et al also successfully used a similar technique implanting a tape suture construct with anchors in a clinical setting for the treatment of symphyseal instability, however, with a laparoscopic approach [30]. Yet, this laparoscopic procedure requires additional surgical access points to enter the abdominal cavity, in our opinion exceeding the necessary invasiveness. To our minds, fluoroscopy is sufficient for the identification of anatomical landmarks and correct placement of the anchors. Hence, we think that a laparoscopy does not provide any added value to the surgical procedure but instead tends to increase the invasiveness.

Considering the second question of this study, few authors have described promising biomechanical results using flexible implants in a criss-cross technique [29, 30], however, to our knowledge there is no study that in addition evaluates a triangle technique.

In our study, we found no significant difference considering the biomechanical stability of both techniques (*p* > 0.05). The triangle technique, however, requires optimal anchor placement and tension: in case of misplacement or minimal dislocation of one anchor, the entire constructs stability is potentially at stake. The criss-cross technique with its four anchors and two triangles is possibly able to compensate the loss of tension in one triangle with the second triangle. Nevertheless, the triangle technique requires one anchor and one drilling hole less than the criss-cross technique and can potentially be performed with one FiberTape^®^ only, saving implant cost, operating time and reducing potential surgical complications.

In addition, the use of absorbable anchor systems potentially allows the dispense of long-term implants with the need for operative removal.

Long-term and especially clinical studies, however, need to follow to confirm or refute the biomechanical findings of both techniques in this study.

In this study, the use of identical synthetic bone models eliminates potential confounding interindividual variability such as anatomical variations or differences in the bone structure, however, lacks the capability of fully representing biological human pelvises. Since the biomechanical influence of the posterior pelvic ring and other soft tissues, such as ligaments, was excluded, this study allows the solitary evaluation of the investigated constructs on the symphyseal joint only. Yet, further studies should include the investigation of these constructs and techniques in full pelvis and cadaver models to confirm the biomechanical feasibility and transferability in a clinical setting.

The forces applied to the symphysis during this study were based on prior studies of Meissner et al and Walheim et al. These authors studied the physiological mobility of the pubic symphysis and the concomitant impacting forces [8, 13, 31]. Accordingly, when applying physiological forces to the isolated symphysis with the biomechanically significant posterior pelvic ring previously excluded, the symphysis is not able to bear the entire load and will inevitably rupture, independently from any applied osteosynthesis [13]. Consequently, the authors suggested a maximum of + 75 N and a minimum of −75 N during cyclic loading which was adopted in this study in order to ensure a reasonable biomechanical comparison of the tested osteosynthesis methods [13].

Finally, considering the biomechanical properties and the promising results of the tape suture construct in this study, one can possibly use this osteosynthesis not only for the treatment of open book fractures, but also for patients suffering from other pelvic instabilities, such as pelvic girdle pain and symphysis pubis dysfunction. This symptom complex represents an underestimated but widespread problem with so far only few investigations on minimally invasive treatment options. Further studies are planned by this study group to test the feasibility of the semi rigid implants already successfully examined in this study for the treatment of pelvic girdle pain and symphysis pubis dysfunction.

## Conclusion

In this study, we not only confirmed symphyseal plating to be a rigid osteosynthesis almost entirely compromising the mobility of the symphyseal joint for the treatment of open book fractures, but were also able to identify the modified SpeedBridge^™^ as a semi-rigid implant to provide sufficient biomechanical stability to the symphyseal joint while still maintaining a minimum of symphyseal movement. This way, the tape suture construct may ensure adequate healing of the injured joint while preventing an iatrogenic arthrodesis.

We found no biomechanical difference between the criss-cross and the triangle technique.
